# Case of Abcess of Spleen, with Perforation of Large Intestine

**Published:** 1878-01

**Authors:** Geo. E. Brown

**Affiliations:** Resident Physician at St. Luke’s Hospital


### Case of Abscess of Spleen, with Perforation of Large Intestine.

By Dr. Geo. E. Brown,

(*Resident Physician at St. Luke's Hospital.*)

Mrs. F., æt. 31.—Her father died from phthisis pulmonalis. When she was 12 years old had typhoid fever, as was supposed, since which time she had had an icteric hue of the complexion, and almost a constant pain in the region of the spleen, aggravated at times.

Came to this country four years ago, and had recurring chills since then, regarding which not enough could be learned to enable us to say positively that they were malarial, though, at first, as nearly as could be learned, the chills recurred every second day, but when she came under our observation, Sept. 27th last, they obeyed no such regularity.

Last winter she was troubled with cough, and was thought to be the subject of phthisis.

Last June she gave birth to her first child, having been married but about one year; the placenta was retained four days, and then taken away piece-meal, after considerable loss of blood.

For two months after this she was very low, but, recuperating to some extent, her friends thought it advisable to remove her to St. Luke's, where she could be under constant medical attendance. When she was admitted to the hospital she had a peculiar yellowish cachectic appearance of the countenance; was extremely emaciated and anæmic.

Treatment, for a time, seemed to benefit her; then she grew worse, having irregularly recurring chills, constant though moderate pyrexia, and profuse nocturnal perspirations.

Oct. 3rd, had four severe hemorrhages from the bowels, coincidently with which the enlargement of the spleen—which I neglected to mention in the proper place—subsided. Unaccountable as it was, she was better after the hemorrhages, and began to improve. However, the improvement was only temporary.

In a few days profuse fluid or semi-fluid discharge from the bowels came on, when was discovered, for the first time, the fact that these evacuations were largely made up of *pus*.

The true nature of the case did not even then dawn upon us; indeed it did not until our ocular sense revealed it to us at the post-mortem examination. The patient rapidly sank, and died Oct. 15th.

The following is a synopsis of the post-mortem:

Extensive pleuritic adhesions over left lung, pleura thickened, and pleural cavity half full of dirty, yellowish-looking fluid; hypostatic congestion of the lower lobe of right lung.

These thoracic troubles, it may be remarked, were entirely latent excepting a cough that she had *last winter*.

Abdominal cavity: Peritoneal adhesions over left side, binding the descending colon to the spleen; some inflammatory serum in cavity of peritoneum.

Spleen disorganized and the seat of large cavities, and disseminated smaller ones, filled with a darkish-gray, purulent, and extremely offensive material; cretaceous concretions in walls of cavities.

Descending colon adjacent to spleen perforated by ulceration, allowing a communication between the cavity in the spleen and the intestine.

Liver enlarged, and centers of portal lobules congested; ulceration of os uteri.

The ovaries were disorganized.

The explanation of various phenomena of this patient's illness is too manifest to require comment.

